# Response to Grisedale and Van Daal: comparison of STR profiling from low template DNA extracts with and without the consensus profiling method

**DOI:** 10.1186/2041-2223-4-1

**Published:** 2013-01-03

**Authors:** Bas Kokshoorn, Bart J Blankers

**Affiliations:** 1 Department of Human Biological Traces, Netherlands Forensic Institute, P.O. Box 24044, The Hague, NL-2490AA, the Netherlands

## Abstract

In a recent contribution to this journal Grisedale and Van Daal concluded that a single STR analysis of all available template DNA is to be preferred over replicate analyses and a consensus approach when analyzing low template DNA samples. A single STR analysis approach does not allow for an assessment of the validity of the resulting DNA profile. We argue that the use of replicate amplifications is the best way to objectively quantify the extent of the stochastic variation in the data. By applying consensus methodology and/or a probabilistic model, the interpretation of the data will therefore be more objective and reliable.

## 

Advances in forensic DNA typing over the past 25 years have made it possible to obtain DNA profiles from samples with increasingly small amounts of DNA. The consecutive interpretation of these DNA profiles can be very complex due to stochastic effects. These effects, like allele drop out, allele drop in (sporadic contamination and/or elevated stutters) and heterozygous peak imbalance, are inherent to low template DNA (LT-DNA) profiling. One of the major challenges when interpreting LT-DNA results is to distinguish artifact peaks from actual alleles derived from DNA present in the sample. Statistical models that incorporate the probabilities of stochastic effects to provide an evidential value to DNA profile comparisons have previously been described [1,2]. Such probabilistic models are currently implemented in casework while further development and exploration are continuing. This is in part because, as Gill and colleagues [3] stated, ‘There is currently no statistical model that incorporates all these parameters simultaneously. In this respect all existing models must be considered incomplete (indeed we must consider that a complete model is unattainable)’. In anticipation of further development and implementation of these statistical models, the consensus method (biological model) was proposed as a conservative means to interpret LT-DNA profiles [4]. The optimal way to apply this model and the use for mock case samples were extensively studied by Benschop and colleagues [5-7]. In their recent contribution to *Investigative Genetics*, Grisedale and Van Daal report on the comparison of STR profiling from LT-DNA extracts with and without the consensus profiling method [8]. Based on their studies they conclude that a single STR analysis of all available template DNA is to be preferred over replicate analyses and a consensus approach.

There are issues that the authors fail to address in their study, which in our view are fundamental to case work. We therefore do not feel that this general conclusion can be drawn based on the data presented in the study that was published.

The major issue that is not sufficiently addressed is reliability of the obtained results; that is, the validity of the peaks observed in the DNA profile. The extent to which the results can be influenced by stochastic effects is usually assessed by applying a stochastic threshold. This threshold has previously been defined as an arbitrary template amount of 100 or 200 pg per sample [4]. This definition of stochastic threshold, however, is in disuse [9]. Samples containing more than 100 or 200 pg are not devoid of stochastic effects, while newer generation DNA typing systems with increased sensitivity generally yield DNA profiles with few stochastic effects for samples with lower amounts of DNA. Since a threshold based on the total amount of DNA in a sample also holds no value for mixtures (since the amount of DNA per donor is less), a relative fluorescence units (RFU) threshold applied to the heights of peaks in the electropherogram is regarded to be far more informative. Such a RFU stochastic threshold should be determined for each STR analysis system and for each amplification and post-PCR analysis protocol. For instance, the Netherlands Forensic Institute has validated and implemented the Next Generation Multiplex analysis system [10]. Standard analysis is carried out with 29 amplification cycles and injection setting of 5 seconds at 3kV. The stochastic threshold determined for these settings (that is, <1% probability of drop out of heterozygous alleles) is 175 RFU. For enhanced detection other injection settings may be used (that is, 15 seconds at 3kV), for which a stochastic threshold of 400 RFU has been determined.

Based on an initial DNA analysis of the sample the best approach for subsequent DNA analyses can be established. Only after this analysis is it clear whether the sample is single source or contains DNA from multiple donors. The relative contributions of donors to the sample can also be estimated based on their relative peak heights. A single STR analysis of all DNA template (as suggested by Grisedale and Van Daal) is therefore only indicated when the resulting DNA analysis will yield a DNA profile in which all peaks of all donors are above the specified stochastic threshold.

Forensic practice shows that most low template casework samples represent mixtures of DNA from two or more donors, often in unequal mixture ratios. In these cases, it will be nearly impossible to differentiate between stochastic effects and alleles of (minor) contributors based on a single DNA profile, even when applying a stochastic threshold. Replicating the DNA analysis and applying a consensus model is then a helpful (if not essential) tool to assess the extent of stochastic effects. Even when a probabilistic model is applied instead of a consensus approach, replicate analyses will better account for the uncertainties regarding the stochastic effects in the underlying biochemical processes [11,12].

In our experience, a single analysis of all available template material in LT-DNA samples, as proposed by Grisedale and Van Daal, will generally not yield a DNA profile of sufficient quality (that is, with all alleles of all donors above the stochastic threshold). Therefore, even though the approach forwarded by Grisedale and Van Daal may yield more peaks in the DNA profile, the uncertainty about their validity will make subsequent interpretation difficult and the inferences less reliable. A consensus approach may yield fewer peaks (because of more allele and/or locus drop out) but the interpretation of the remaining peaks will be less equivocal and more objective.

Essentially, one should distinguish between the quantity and the quality of information. In our view the latter should be preferred in most – if not all – cases.

## Abbreviations

LT-DNA: low template DNA; RFU: relative fluorescence units; STR: short tandem repeat.

## Competing interests

The authors declare that they have no competing interests.

## Authors’ contribution

Both authors contributed equally and have read and approved the final manuscript.
